# Sexuality and Treatment Adherence in Depressed Patients Treated with Antidepressants: A Cross-Sectional Study

**DOI:** 10.1192/j.eurpsy.2026.12106

**Published:** 2026-07-31

**Authors:** H. Zizi, R. Rahoua, Z. Ennaciri, I. Adali, F. Manoudi

**Affiliations:** Ibn Nafis hospital, Psychiatry, Mohammed VI university hospital, Marrakech, Morocco

## Abstract

**Introduction:**

Sexual dysfunction is a frequent adverse effect of antidepressants, particularly selective serotonin reuptake inhibitors (SSRIs) and serotonin-norepinephrine reuptake inhibitors (SNRIs). It is often underestimated and may compromise treatment adherence. Few studies have examined both its prevalence and its impact on adherence in depressed patients.

**Objectives:**

To assess the prevalence of sexual dysfunction in patients treated with antidepressants and to analyze its relationship with treatment adherence, with a focus on differences between drug classes.

**Methods:**

We conducted a descriptive and analytical cross-sectional study over one month including 100 psychiatric outpatients aged 18–65 years, diagnosed with a major depressive episode, and treated with antidepressants for at least four weeks. Patients with a history of sexual dysfunction, severe somatic comorbidities, or psychoactive substance use affecting sexuality were excluded. Sexual function was assessed using the Arizona Sexual Experience Scale (ASEX), adherence with the Morisky Medication Adherence Scale (MMAS-8), and depression severity with the Montgomery–Åsberg Depression Rating Scale (MADRS).

**Results:**

The sample included 60 women and 40 men, with a mean age of 38.5 ± 12.2 years. Treatments were mainly SSRIs (65%), followed by SNRIs (25%) and other antidepressants (10%).

The overall prevalence of sexual dysfunction was 42%, mainly affecting orgasm (50%), sexual satisfaction (48%), and desire (45%). The mean ASEX score was 19.0 ± 5.2.

Treatment adherence, assessed by the MMAS-8, was insufficient in 35% of patients. Sexual dysfunction was significantly associated with poorer adherence (55% vs 22%, p < 0.01). A negative correlation was found between ASEX and MMAS-8 scores (r = –0.42, p < 0.01), indicating that greater severity of sexual dysfunction was linked to lower adherence.

Sexual dysfunction prevalence varied according to drug class: 50% with SSRIs, 44% with SNRIs, and only 10% with bupropion/mirtazapine (p < 0.01). Adherence followed the same trend, with poorer adherence under SSRIs (40%) and SNRIs (36%) compared with atypical antidepressants (10%).

**Conclusions:**

Sexual dysfunction is common among patients receiving antidepressants and is strongly associated with non-adherence, particularly under SSRIs and SNRIs. Routine screening and appropriate management are essential to improve adherence and optimize depression outcomes.

**Disclosure of Interest:**

None Declared

